# Supplementary material for: Accuracy of dipole source reconstruction in the 3-layer BEM model against the 5-layer BEM-FMM model
Source: bioRxiv. 2024 May 21:2024.05.17.594750. Preprint. [Version 1] doi: 10.1101/2024.05.17.594750 (PMC11142039; doi:10.1101/2024.05.17.594750)
Supplement: Supplement 1 [file media-1.pdf]

Supplement A: Tables

1 Tables of results per dipole position

Here we include tables with results per dipole separated among those experiments using forward simulated data from FreeSurfer and Headreco segmentation meshes. We also include the 3-layer model comparison data, where the forward solution has been computed using the 3-shell decimated models. Two important observations can be made:

(i) The variability of dipole fits from EEG data generated with Headreco models is larger than that generated with FreeSurfer models.

(ii) The EEG data generated without AMR has numerical errors, reflected on the fitting results with larger errors in the dipole fit, and higher variabilities.

1.1 FreeSurfer segmentation results

Table 1: AMR — FreeSurfer.
Forward model: FreeSurfer with 7 high-resolution compartments computed with BEM-FMM+AMR. Inverse model: Decimated FreeSurfer shells with 3 compartments of circa 14 000 triangles.

Dipole	Dist-mm	Angle-deg	Residual-Variance	Total-AMR-steps
dip1	4.37	8.70	5.96×10^{-4}	5.56
dip2	4.38	14.20	5.49×10^{-4}	5.64
dip3	2.47	14.52	9.59×10^{-4}	4.29
dip4	4.12	18.07	2.474×10^{-3}	8.60

(a) Means

Dipole	Dist-mm	Angle-deg	Residual-Variance	Total-AMR-steps
dip1	1.55	4.50	8.22×10^{-4}	2.49
dip2	1.27	7.51	3.10×10^{-4}	2.05
dip3	1.07	7.16	6.75×10^{-4}	1.85
dip4	1.87	9.27	1.921×10^{-3}	4.21

(b) Standard Deviations

The following table includes the results of the dipole fitting without using AMR in the computation of the forward solution. Notice the dramatic decrease in fitting accuracy, and the higher variability, both of which are caused by numerical inaccuracies.

Table 2: non-AMR — FreeSurfer. Forward model: FreeSurfer with 7 high-resolution compartments computed with BEM-FMM (no AMR). Inverse model: Decimated FreeSurfer shells with 3 compartments of circa 14 000 triangles.

Dipole	Dist-mm	Angle-deg	Residual-Variance
dip1	8.45	22.66	4.918×10^{-3}
dip2	5.38	13.72	1.496×10^{-3}
dip3	4.29	19.80	2.913×10^{-3}
dip4	14.78	81.03	9.998×10^{-3}

(a) means

Dipole	Dist-mm	Angle-deg	Residual-Variance
dip1	8.27	15.09	3.955×10^{-3}
dip2	2.65	8.14	1.534×10^{-3}
dip3	3.18	13.59	2.833×10^{-3}
dip4	3.71	55.42	4.902×10^{-3}

(b) Standard Deviations

1.2 Headreco segmentation results

Table 3: AMR — Headreco

Dipole	Dist-mm	Angle-deg	Residual-Variance	Total-AMR-steps
dip1	3.91	8.72	7.84×10^{-4}	6.80
dip2	5.57	14.45	1.160×10^{-3}	5.67
dip3	2.37	14.36	1.008×10^{-3}	4.89
dip4	6.73	12.10	1.099×10^{-3}	7.93

(a) AMR means — Headreco

Dipole	Dist-mm	Angle-deg	Residual-Variance	Total-AMR-steps
dip1	1.73	4.95	3.60×10^{-4}	3.66
dip2	2.03	9.90	5.56×10^{-4}	2.17
dip3	1.16	7.39	4.49×10^{-4}	3.00
dip4	4.74	5.93	7.31×10^{-4}	4.92

(b) AMR Standard Deviations — Headreco

Table 4: non-AMR — Headreco

Dipole	Dist-mm	Angle-deg	Residual-Variance
dip1	10.49	27.01	7.340×10^{-3}
dip2	8.37	25.54	3.592×10^{-3}
dip3	3.66	17.58	2.423×10^{-3}
dip4	5.31	19.05	3.002×10^{-3}

(a) non-AMR means — Headreco

Dipole	Dist-mm	Angle-deg	Residual-Variance
dip1	8.71	20.30	6.596×10^{-3}
dip2	5.58	41.87	3.033×10^{-3}
dip3	2.67	10.16	3.179×10^{-3}
dip4	3.09	13.05	4.325×10^{-3}

(b) non-AMR Standard Deviations — Headreco

1.3 3-layer model comparison results

Table 5: AMR results per dipole — 3-layer model comparison

Dipole	Dist-mm	Angle-deg	Residual-Variance	Total-AMR-steps
dip1	1.02	0.57	5.5×10^{-5}	2.24
dip2	2.40	1.41	3.23×10^{-4}	3.31
dip3	0.39	0.60	2.0×10^{-5}	2.96
dip4	0.42	0.42	1.3×10^{-5}	2.78

(a) AMR Averages per dipole — 3-layer model comparison

Dipole	Dist-mm	Angle-deg	Residual-Variance	Total-AMR-steps
dip1	0.27	0.36	2.8×10^{-5}	0.43
dip2	0.67	1.32	2.62×10^{-4}	1.02
dip3	0.16	0.44	1.2×10^{-5}	1.15
dip4	0.19	0.32	9×10^{-6}	1.02

(b) AMR Standard deviations per dipole — 3-layer model comparison

Table 6: No-AMR Averages per dipole — 3-layer model comparison

Dipole	Dist-mm	Angle-deg	Residual-Variance
`dip1`	1.59	2.60	2.93×10^{-4}
`dip2`	2.08	2.77	9.10×10^{-4}
`dip3`	3.06	1.62	1.79×10^{-4}
`dip4`	3.04	1.18	2.70×10^{-4}

(a) No-AMR Averages per dipole — 3-layer model comparison

Dipole	Dist-mm	Angle-deg	Residual-Variance
`dip1`	1.00	2.57	2.50×10^{-4}
`dip2`	0.88	2.71	5.56×10^{-4}
`dip3`	2.10	1.77	1.50×10^{-4}
`dip4`	2.18	0.94	3.43×10^{-4}

(b) No-AMR Standard deviations per dipole — 3-layer model comparison

1.4 Combined AMR results: FreeSurfer+Headreco

Table 7: AMR — all 7-shell models

Dipole	Dist-mm	Angle-deg	Residual-Variance	Total-AMR-steps
`dip1`	4.14	8.71	6.90×10^{-4}	6.18
`dip2`	4.98	14.33	8.54×10^{-4}	5.66
`dip3`	2.42	14.44	9.83×10^{-4}	4.59
`dip4`	5.42	15.08	1.787×10^{-3}	8.27

(a) AMR means — all 7-shell models

Dipole	Dist-mm	Angle-deg	Residual-Variance	Total-AMR-steps
`dip1`	1.65	4.70	6.38×10^{-4}	3.17
`dip2`	1.78	8.74	5.43×10^{-4}	2.10
`dip3`	1.11	7.24	5.70×10^{-4}	2.50
`dip4`	3.82	8.30	1.602×10^{-3}	4.56

(b) AMR Standard Deviations — all 7-shell models

1.5 Combined Non-AMR results: FreeSurfer+Headreco

Table 8: Non-AMR results per dipole — all 7-shell models

Dipole	Dist-mm	Angle-deg	Residual-Variance
`dip1`	9.47	24.84	6.129×10^{-3}
`dip2`	6.88	19.63	2.544×10^{-3}
`dip3`	3.97	18.69	2.668×10^{-3}
`dip4`	10.04	50.04	6.500×10^{-3}

(a) Non-AMR means — all 7-shell models

Dipole	Dist-mm	Angle-deg	Residual-Variance
`dip1`	9.47	24.84	6.129×10^{-3}
`dip2`	6.88	19.63	2.544×10^{-3}
`dip3`	3.97	18.69	2.668×10^{-3}
`dip4`	10.04	50.04	6.500×10^{-3}

(b) Non-AMR standard deviations — all 7-shell models

2 Tables of results per model and dipole

2.1 FreeSurfer segmentation results

Table 9: AMR — FreeSurfer

Model	Dipole	Dist-mm	Angle-deg	Residual-Variance	Total-AMR-steps
SimNIBS7	dip1	4.77	10.41	5.04×10^{-4}	6.33
SimNIBS7	dip2	4.66	15.39	4.92×10^{-4}	6.07
SimNIBS7	dip3	2.88	17.42	9.64×10^{-4}	4.93
SimNIBS7	dip4	5.00	22.12	3.38×10^{-3}	8.93
VWB7	dip1	5.06	9.94	8.37×10^{-4}	5.40
VWB7	dip2	4.93	17.24	5.84×10^{-4}	6.07
VWB7	dip3	2.96	16.87	1.01×10^{-3}	4.53
VWB7	dip4	4.93	20.25	2.81×10^{-3}	8.67
IT'IS7	dip1	3.26	5.75	4.47×10^{-4}	4.93
IT'IS7	dip2	3.57	9.96	5.70×10^{-4}	4.80
IT'IS7	dip3	1.56	9.28	9.04×10^{-4}	3.40
IT'IS7	dip4	2.43	11.82	1.23×10^{-3}	8.20

(a) AMR means — FreeSurfer

Model	Dipole	Dist-mm	Angle-deg	Residual-Variance	Total-AMR-steps
SimNIBS7	dip1	1.19	4.44	3.07×10^{-4}	2.66
SimNIBS7	dip2	1.05	7.42	2.53×10^{-4}	2.05
SimNIBS7	dip3	0.94	5.47	7.79×10^{-4}	1.87
SimNIBS7	dip4	1.84	9.99	1.94×10^{-3}	5.96
VWB7	dip1	1.89	4.21	1.38×10^{-3}	2.90
VWB7	dip2	1.50	7.66	2.99×10^{-4}	2.02
VWB7	dip3	1.01	6.48	8.05×10^{-4}	1.88
VWB7	dip4	1.45	8.73	2.13×10^{-3}	4.22
IT'IS7	dip1	0.75	3.48	1.69×10^{-4}	1.71
IT'IS7	dip2	0.77	5.73	3.77×10^{-4}	1.93
IT'IS7	dip3	0.64	6.71	4.11×10^{-4}	1.55
IT'IS7	dip4	0.91	5.44	7.98×10^{-4}	1.42

(b) AMR Standard Deviations — FreeSurfer

Table 10: non-AMR — FreeSurfer

Model	Dipole	Dist-mm	Angle-deg	Residual-Variance
SimNIBS7	dip1	9.55	26.10	4.63×10^{-3}
SimNIBS7	dip2	5.89	15.25	1.75×10^{-3}
SimNIBS7	dip3	5.52	25.36	3.22×10^{-3}
SimNIBS7	dip4	15.87	89.49	9.95×10^{-3}
VWB7	dip1	10.16	26.68	5.37×10^{-3}
VWB7	dip2	6.18	15.92	1.57×10^{-3}
VWB7	dip3	5.16	21.90	3.16×10^{-3}
VWB7	dip4	15.34	78.53	8.89×10^{-3}
IT'IS7	dip1	5.65	15.20	4.74×10^{-3}
IT'IS7	dip2	4.07	10.00	1.18×10^{-3}
IT'IS7	dip3	2.18	12.14	2.36×10^{-3}
IT'IS7	dip4	13.12	75.06	1.12×10^{-2}

(a) non-AMR means — FreeSurfer

Model	Dipole	Dist-mm	Angle-deg	Residual-Variance
SimNIBS7	dip1	9.40	15.46	2.87×10^{-3}
SimNIBS7	dip2	3.31	8.82	1.72×10^{-3}
SimNIBS7	dip3	3.57	17.43	2.31×10^{-3}
SimNIBS7	dip4	3.04	50.93	4.73×10^{-3}
VWB7	dip1	9.14	16.49	3.66×10^{-3}
VWB7	dip2	2.71	8.41	1.62×10^{-3}
VWB7	dip3	3.15	9.94	3.31×10^{-3}
VWB7	dip4	3.71	61.10	3.32×10^{-3}
IT'IS7	dip1	5.48	10.72	5.21×10^{-3}
IT'IS7	dip2	0.97	6.11	1.27×10^{-3}
IT'IS7	dip3	1.37	8.79	2.90×10^{-3}
IT'IS7	dip4	3.95	56.60	6.27×10^{-3}

(b) non-AMR Standard Deviations — FreeSurfer

2.2 Headreco segmentation results

Table 11: AMR — Headreco

Model	Dipole	Dist-mm	Angle-deg	Residual-Variance	Total-AMR-steps
SimNIBS7	dip1	3.81	10.29	9.46×10^{-4}	7.93
SimNIBS7	dip2	6.18	16.16	1.26×10^{-3}	6.40
SimNIBS7	dip3	2.87	17.59	1.20×10^{-3}	5.60
SimNIBS7	dip4	9.14	13.93	1.30×10^{-3}	9.60
VWB7	dip1	3.86	10.14	9.13×10^{-4}	7.13
VWB7	dip2	6.12	16.35	1.27×10^{-3}	6.33
VWB7	dip3	2.85	17.72	1.18×10^{-3}	5.13
VWB7	dip4	8.67	13.50	1.25×10^{-3}	8.53
IT'IS7	dip1	4.06	5.75	4.92×10^{-4}	5.33
IT'IS7	dip2	4.41	10.86	9.54×10^{-4}	4.27
IT'IS7	dip3	1.39	7.77	6.44×10^{-4}	3.93
IT'IS7	dip4	2.38	8.87	7.41×10^{-4}	5.67

(a) AMR means — Headreco

Model	Dipole	Dist-mm	Angle-deg	Residual-Variance	Total-AMR-steps
SimNIBS7	dip1	2.04	5.36	3.64×10^{-4}	4.92
SimNIBS7	dip2	2.01	10.44	5.32×10^{-4}	2.03
SimNIBS7	dip3	1.09	6.52	4.28×10^{-4}	3.72
SimNIBS7	dip4	4.45	6.29	8.01×10^{-4}	6.15
VWB7	dip1	1.95	5.21	3.06×10^{-4}	3.18
VWB7	dip2	2.01	10.30	5.58×10^{-4}	1.91
VWB7	dip3	1.08	6.95	4.41×10^{-4}	3.09
VWB7	dip4	4.40	6.22	7.54×10^{-4}	4.79
IT'IS7	dip1	1.17	2.62	2.10×10^{-4}	1.99
IT'IS7	dip2	1.62	8.51	5.55×10^{-4}	1.98
IT'IS7	dip3	0.56	3.33	2.07×10^{-4}	1.83
IT'IS7	dip4	0.97	3.94	5.13×10^{-4}	2.53

(b) AMR Standard Deviations — Headreco

Table 12: non-AMR — Headreco

Model	Dipole	Dist-mm	Angle-deg	Residual-Variance
SimNIBS7	dip1	12.12	31.67	8.09×10^{-3}
SimNIBS7	dip2	10.34	27.00	4.28×10^{-3}
SimNIBS7	dip3	4.88	22.05	3.13×10^{-3}
SimNIBS7	dip4	6.07	21.47	3.70×10^{-3}
VWB7	dip1	11.63	30.62	7.72×10^{-3}
VWB7	dip2	9.69	27.16	4.03×10^{-3}
VWB7	dip3	4.44	21.40	2.82×10^{-3}
VWB7	dip4	5.84	20.43	3.47×10^{-3}
IT'IS7	dip1	7.73	18.75	6.21×10^{-3}
IT'IS7	dip2	5.10	22.48	2.46×10^{-3}
IT'IS7	dip3	1.66	9.27	1.32×10^{-3}
IT'IS7	dip4	4.03	15.25	1.83×10^{-3}

(a) non-AMR means — Headreco

Model	Dipole	Dist-mm	Angle-deg	Residual-Variance
SimNIBS7	dip1	9.38	21.05	6.58×10^{-3}
SimNIBS7	dip2	6.35	42.69	3.21×10^{-3}
SimNIBS7	dip3	2.93	10.53	3.78×10^{-3}
SimNIBS7	dip4	3.27	14.03	5.20×10^{-3}
VWB7	dip1	9.31	20.57	6.44×10^{-3}
VWB7	dip2	6.01	42.52	3.21×10^{-3}
VWB7	dip3	2.56	9.20	3.59×10^{-3}
VWB7	dip4	3.05	13.49	4.91×10^{-3}
IT'IS7	dip1	7.17	17.86	7.06×10^{-3}
IT'IS7	dip2	1.99	43.19	2.50×10^{-3}
IT'IS7	dip3	0.87	4.25	1.60×10^{-3}
IT'IS7	dip4	2.70	11.51	2.32×10^{-3}

(b) non-AMR Standard Deviations — Headreco

2.3 Calibration results

Table 13: AMR — Calibration

Model	Dipole	Dist-mm	Angle-deg	Residual-Variance	Total-AMR-steps
SimNIBS3	dip1	1.14	0.73	6.61×10^{-5}	2.40
SimNIBS3	dip2	2.85	1.54	2.95×10^{-4}	3.87
SimNIBS3	dip3	0.38	0.85	2.24×10^{-5}	3.20
SimNIBS3	dip4	0.52	0.59	1.79×10^{-5}	3.00
VWB3	dip1	1.14	0.69	6.42×10^{-5}	2.33
VWB3	dip2	2.75	1.77	3.89×10^{-4}	3.80
VWB3	dip3	0.38	0.78	2.18×10^{-5}	3.13
VWB3	dip4	0.48	0.54	1.61×10^{-5}	2.93
IT'IS3	dip1	0.79	0.30	3.49×10^{-5}	2.00
IT'IS3	dip2	1.60	0.92	2.86×10^{-4}	2.27
IT'IS3	dip3	0.40	0.18	1.68×10^{-5}	2.53
IT'IS3	dip4	0.25	0.13	5.08×10^{-6}	2.40

(a) AMR means — Calibration

Model	Dipole	Dist-mm	Angle-deg	Residual-Variance	Total-AMR-steps
SimNIBS3	dip1	0.25	0.36	2.87×10^{-5}	0.51
SimNIBS3	dip2	0.36	1.36	1.77×10^{-4}	0.64
SimNIBS3	dip3	0.18	0.40	1.30×10^{-5}	1.26
SimNIBS3	dip4	0.19	0.32	8.24×10^{-6}	1.07
VWB3	dip1	0.24	0.36	2.76×10^{-5}	0.49
VWB3	dip2	0.40	1.67	3.74×10^{-4}	0.68
VWB3	dip3	0.19	0.38	1.31×10^{-5}	1.30
VWB3	dip4	0.18	0.28	7.66×10^{-6}	1.16
IT'IS3	dip1	0.13	0.16	1.58×10^{-5}	0.00
IT'IS3	dip2	0.30	0.67	1.96×10^{-4}	0.80
IT'IS3	dip3	0.12	0.13	1.05×10^{-5}	0.74
IT'IS3	dip4	0.06	0.05	2.46×10^{-6}	0.74

(b) AMR Standard Deviations — Calibration

Table 14: non-AMR — Calibration

Model	Dipole	Dist-mm	Angle-deg	Residual-Variance
SimNIBS3	dip1	2.22	3.92	4.41×10^{-4}
SimNIBS3	dip2	2.48	3.76	1.22×10^{-3}
SimNIBS3	dip3	4.67	2.46	2.78×10^{-4}
SimNIBS3	dip4	4.64	1.75	4.32×10^{-4}
VWB3	dip1	2.01	3.54	3.89×10^{-4}
VWB3	dip2	2.41	3.59	1.13×10^{-3}
VWB3	dip3	4.31	2.21	2.41×10^{-4}
VWB3	dip4	4.25	1.58	3.67×10^{-4}
IT'IS3	dip1	0.55	0.33	4.94×10^{-5}
IT'IS3	dip2	1.35	0.97	3.78×10^{-4}
IT'IS3	dip3	0.21	0.19	1.84×10^{-5}
IT'IS3	dip4	0.21	0.21	9.59×10^{-6}

(a) non-AMR means — Calibration

Model	Dipole	Dist-mm	Angle-deg	Residual-Variance
SimNIBS3	dip1	0.86	2.59	2.35×10^{-4}
SimNIBS3	dip2	0.83	2.95	4.66×10^{-4}
SimNIBS3	dip3	0.64	1.89	1.27×10^{-4}
SimNIBS3	dip4	1.06	0.82	3.92×10^{-4}
VWB3	dip1	0.78	2.38	2.10×10^{-4}
VWB3	dip2	0.84	2.92	4.33×10^{-4}
VWB3	dip3	0.59	1.72	1.10×10^{-4}
VWB3	dip4	0.97	0.74	3.25×10^{-4}
IT'IS3	dip1	0.18	0.26	1.77×10^{-5}
IT'IS3	dip2	0.41	0.76	3.24×10^{-4}
IT'IS3	dip3	0.08	0.14	1.18×10^{-5}
IT'IS3	dip4	0.10	0.10	4.31×10^{-6}

(b) non-AMR Standard Deviations — Calibration
